# Evaluating the role of IDO1 macrophages in immunotherapy using scRNA-seq and bulk-seq in colorectal cancer

**DOI:** 10.3389/fimmu.2022.1006501

**Published:** 2022-09-29

**Authors:** Xingwu Liu, Guanyu Yan, Boyang Xu, Han Yu, Yue An, Mingjun Sun

**Affiliations:** ^1^ Department of Gastroenterology, The First Hospital of China Medical University, Shenyang, China; ^2^ School of Health Management, China Medical University, Shenyang, China; ^3^ Department of Endoscopy, The First Hospital of China Medical University, Shenyang, China

**Keywords:** colorectal cancer (CRC), macrophage, tumor immunity, immunotherapy, single-cell RNA sequencing (scRNA-seq), IDO1

## Abstract

**Background:**

Macrophage infiltration is crucial for colorectal cancer (CRC) immunotherapy. Detailed classification of macrophage subsets will facilitate the selection of patients suitable for immunotherapy. However, the classification of macrophages in CRC is not currently detailed.

**Methods:**

In this study, we combined single-cell RNA sequencing (scRNA-seq) and bulk-seq to analyze patients with colorectal cancer. scRNA-seq data were used to study cell-cell communication and to differentiate immune-infiltrating cells and macrophage subsets. Bulk-seq data were used to further analyze immune infiltration, clinical features, tumor mutational burden, and expression of immune checkpoint molecules in patients with CRC having different macrophage subsets.

**Results:**

Seven macrophage subpopulations were identified, among which indoleamine 2,3 dioxygenase 1 (IDO1) macrophages had the most significant difference in the degree of infiltration among normal, microsatellite-unstable, and microsatellite-stable populations. We then performed gene set variation analysis using 12 marker genes of IDO1 macrophages and divided the patients into two clusters: high-IDO1 macrophages (H-IDO1M) and low-IDO1 macrophages (L-IDO1M). H-IDO1M showed higher infiltration of immune cells, higher expression of immune checkpoints, and less advanced pathological stages than L-IDO1M (p < 0.05).

**Conclusions:**

This study elucidated that IDO1-macrophage-based molecular subtypes can predict the response to immunotherapy in patients with CRC. The results provide new insights into tumor immunity and help in clinical decisions regarding designing effective immunotherapy for these patients.

## Introduction

Colorectal cancer (CRC) is a common cancer with considerable morbidity and mortality rates. For both males and females, the morbidity of CRC ranks third among all cancers, and CRC is the second leading cause of cancer-related deaths worldwide, representing a significant health burden (1). Furthermore, the global burden of CRC is expected to increase by 60% to more than 2.2 million new cases and 1.1 million cancer deaths by 2030 (2).

Conventional treatments for CRC include endoscopy, surgery, radiotherapy, chemotherapy, and traditional Chinese medicine (3–5). However, each of the abovementioned treatments has limitations and is associated with specific adverse effects and complications. For example, 5-Fluorouracil (5-FU) is one of the most commonly used chemotherapeutic drugs for the treatment of CRC; however, a large percentage of patients are resistant to 5-FU (6). With respect to radiotherapy, there is evidence that patients undergoing radiation are more likely to be depressed, distressed, and anxious (7). In addition, although traditional Chinese medicines such as the Gegen Qinlian decoction can modulate the gut microbiota, it is often used as an adjunctive therapy (4, 8). Thus, the development of novel, effective therapeutic approaches is crucial for successfully treating patients with CRC. Immunotherapy has emerged over time as one such treatment. Immunotherapy has successfully achieved long-lasting durable responses in previously difficult-to-treat solid tumors. Immunotherapy also has side effects called immune-related adverse events (irAEs); however, irAEs are usually manageable (9).

CRC is categorized into two major subtypes: deficient mismatch repair (dMMR)/microsatellite instability-high (MSI-H), which accounts for 15% of CRC cases, and microsatellite-stable (MSS)/microsatellite instability-low (MSI-L), which accounts for 85% of cases (10). The high tumor mutational burden (TMB) in dMMR/MSI-H tumors is beneficial for the infiltration of immune cells. Conversely, MSS/MSI-L CRC has a very low TMB, and its infiltration of immune cells is thus minimal. Therefore, patients with CRC with dMMR/MSI-H tumors are more likely to benefit from immunotherapy.

The tumor microenvironment (TME) consists of immune cells, stromal cells, blood/lymphatic vessels, nerve endings, and extracellular matrix. TME has been shown to play a key role in cancer initiation, progression, and treatment. The importance of the TME in the design of new cancer treatment regimes is apparent. As the most dominant component of the TME, immune infiltration is associated with tumor progression and response to immunotherapy (11). Therefore, a comprehensive analysis of immune infiltration in the TME is important for the development of cancer immunotherapies. Single-cell RNA sequencing (scRNA-seq) allows the definition of molecularly distinct cell subpopulations and is a powerful tool for deconstructing the transcriptomes of complex tissues at the single-cell level (12). Using scRNA-seq, it is possible to systematically study cell-cell communication and track the developmental trajectories of distinct cell lineages (13, 14).

Tumor-associated macrophages (TAM) are one of the major immune-infiltrating cell types in the TME, and are generally divided into M1 and M2 macrophages. Both M1 and M2 macrophages are critical in cancer development and metastasis, and can exert a dual influence on cancer based on different activation states. Tumor killing by M1 macrophages is mainly dependent on the production of glucose, reactive oxygen species (ROS), and nitric oxide (NO), the production of which can lead to oncogene activation in nearby epithelia (15, 16). The support of tumor growth by M2 macrophages is mainly dependent on the β-oxidation of fatty acids and the tricarboxylic acid cycle, as well as on the production of polyamines and L-proline (17, 18). In CRC, TAMs can perform multiple functions, including promoting tumor proliferation and metastasis, enhancing angiogenesis, regulating the immunity of the TME, and interacting with the gut microbiota (19–22). Different functions depend on the different phenotypic polarizations of TAMs; therefore, it is of great significance to study their subgroup classifications. However, the classification of macrophage subsets is not currently detailed. This study aims to use scRNA-seq to subdivide macrophage subsets to facilitate the development of cancer immunotherapies.

**Figure 1:**
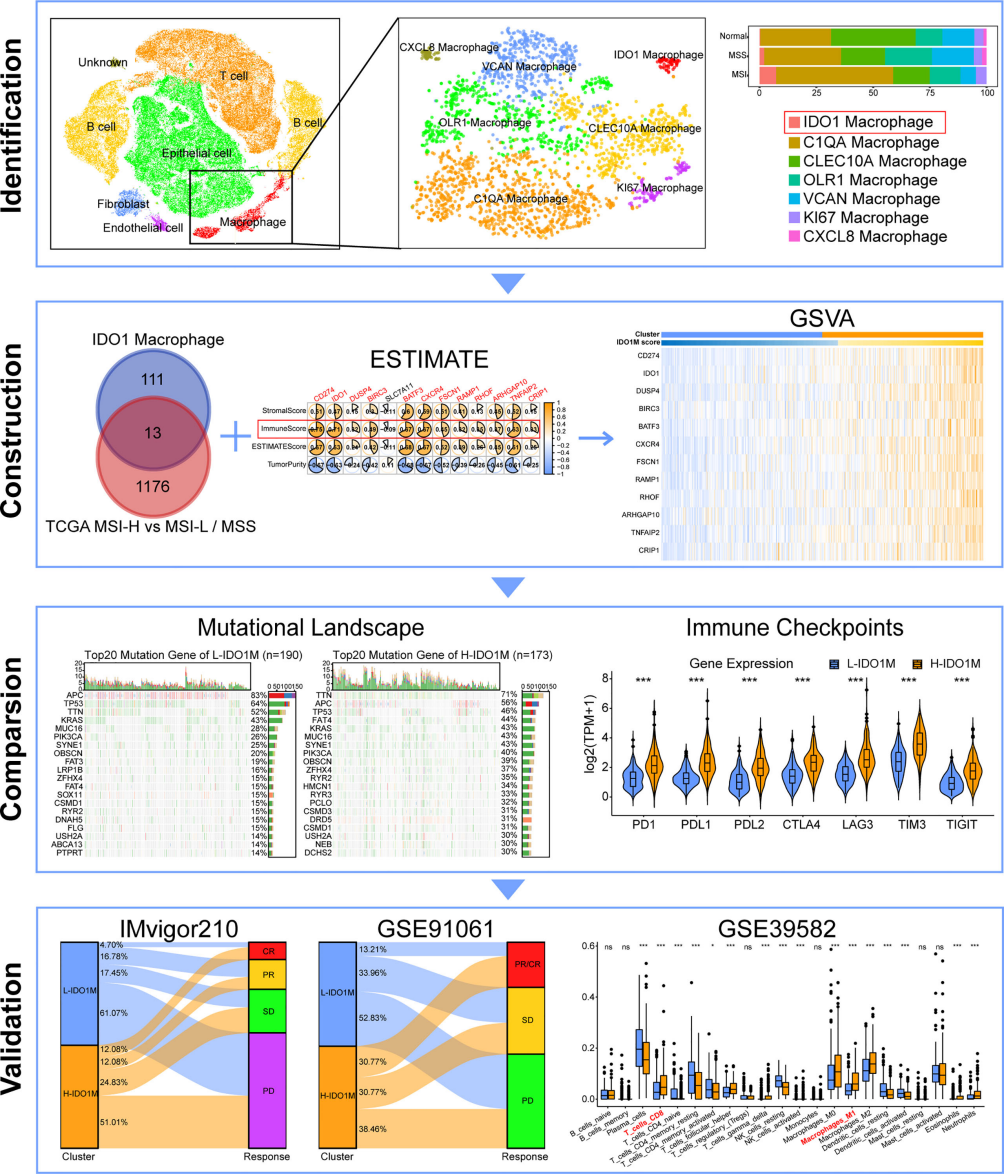
Flowchart of this study. (ns, no significance, *: P < 0.05, ***: P < 0.001).

In this study, single-cell sequencing data were used to classify macrophage subpopulations. We defined a macrophage subset characterized by expression of the immuno-oncological target IDO1. We identified genes related to IDO1 macrophages (IDO1M) and constructed IDO1M scores for each sample using gene set variation analysis (GSVA). We then divided the samples into two clusters based on their IDO1M scores: High-IDO1M (H-IDO1M) and Low-IDO1M (L-IDO1M). H-IDO1M showed higher immune infiltration, TMB, and expression of immune checkpoints than L-IDO1M. Data from the immunotherapy cohorts were used for validation, and the results suggest that the immunotherapeutic effect of H-IDO1M was significantly higher than that of L-IDO1M. The workflow for this study is shown in **Figure 1**. Our results indicate that IDO1 macrophages play an important role in tumor immunity, and patients with CRC having H-IDO1M were more suitable for immunotherapy.

## Materials and methods

### Data acquisition and preprocessing

Single-cell transcriptomic profiles of 28 CRC and 18 adjacent normal tissues were obtained from both GSE166555 and GSE200997 (23, 24). Samples with unknown microsatellite stabilities were excluded from the study. We analyzed the scRNA-seq data using the R package Seurat (25). The data were normalized using the SCTransform method and integrated using the IntegrateData function. The top 3,000 highly variable genes were identified using the SelectIntegrationFeatures function. Principal component analysis (PCA) and t-distributed stochastic neighbor embedding (t-SNE) were applied to reduce the dimensions based on these 3,000 genes. The FindNeighbors and FindClusters functions were used for cell clustering analysis. Transcriptome profiling data from TCGA-COAD (colon adenocarcinoma) were downloaded using the R package TCGAbiolinks (26). Cases with specific MSS/MSI information were also included. Fragments per kilobase of transcript per million mapped reads, or fragments per kilobase million (FPKM) of 426 primary solid tumor samples were converted to transcripts per kilobase million (TPM) for further analyses, and counts were used for differential analysis. Simple nucleotide variation data (MuTect2) of 363 patients with colon adenocarcinoma (COAD) were collected using the cBioPortal (https://www.cbioportal.org/datasets). TMB was calculated based on simple nucleotide variations using the R package MAFtools, defined as the number of mutations per megabase (27). GSE39582, GSE91061, GSE176307, and IMvigor210 were used as validation sets (28–31). The GSE39582, GSE91061 and GSE176307 datasets were obtained from the GEO database (https://www.ncbi.nlm.nih.gov/gds/). IMvigor210 cohort data were obtained from http://research-pub.gene.com/IMvigor210CoreBiologies. The GSE39582 dataset included 519 colon cancer tissues with specific MMR states. Patients with specific immunotherapeutic responses (complete response, CR; partial response, PR; stable disease, SD; and progressive disease, PD) were included in GSE91061 (n = 105), IMvigor210 (n = 298) and GSE176307 (n=87).

### Cell-cell interaction analysis

Cell-cell interaction analysis was performed using the R package CellChat (32). The R package CellChat requires gene expression data from cells as the user input and models the probability of cell-cell communication by integrating gene expression with prior knowledge of the interactions between signaling ligands, receptors and their cofactors. The Secreted Signaling and Cell-Cell Contact human databases were used. Circle and bubble diagrams were used to display the strength of cell-cell communication networks from the target cell cluster to other cell clusters.

### Immune infiltration analysis

The R package ESTIMATE was applied to evaluate the TME of each patient with COAD and then to assign a stromal score (stromal content), immune score (extent of immune cell infiltration), and an ESTIMATE score (synthetic mark of stroma and immune) to quantify tumor purity (33). Single-sample gene set enrichment analysis (ssGSEA) was used to evaluate the gene set levels of immune cells as well as the CD8 T effector (30, 34). We calculated the extent of infiltration of 28 immune cell types according to the expression levels of genes in 28 published gene sets for immune cells using the R package GSVA. CIBERSORT is a deconvolution algorithm used to calculate the proportion of 22 immune cells (35).

### Differential analysis

The FindMarkers function in the Seurat package was used to calculate differentially expressed genes (DEGs) using the Wilcoxon–Mann–Whitney test. To identify the marker genes for each cluster, the cutoff threshold values were adjusted to a p-value < 0.05, log2FoldChange > 3, pct.1 > 0.5, and pct.2 < 0.5. The R package DESeq2 was used for differential analysis of the transcriptome profiling data (36). The threshold values were |log2FoldChange | > 1 and an adjusted p-value < 0.05. The FindMarkers function in the Seurat package was used to calculate differentially expressed genes (DEGs) using Wilcoxon–Mann–Whitney test. To identify the marker genes for each cluster, the cutoff threshold values were adjusted to a p-value < 0.05, log2FoldChange > 1, and pct.1 > 0.4.

### Gene set enrichment analysis

Gene set enrichment analysis (GSEA) was performed using the R package clusterProfiler for functional enrichment (37). Significant enrichment was identified using the normalized enrichment score (|NES| > 1), an adjusted p-value < 0.05, and a q-value < 0.05.

### Gene set variation analysis

pt?>To evaluate the number of IDO1 macrophages in each sample, we calculated scores based on 12 genes related to IDO1 macrophages using the R package GSVA (38). The samples were divided into two clusters according to the median IDO1 macrophage scores: L-IDO1M (low-IDO1 macrophages) and H-IDO1M (high-IDO1 macrophages).

### Statistical analysis

All statistical analyses were conducted using R software (version 4.1.0). The Wilcoxon rank-sum test and Student’s t test were used to compare two groups depending on the results of Shapiro-Wilk test. Correlation analysis was performed using the Spearman’s coefficient. The chi-squared test was used to compare the clinical characteristics in bulk-seq and the proportion of cells in scRNA-seq (Fisher’s exact test was used when required). Survival curves were constructed using the Kaplan-Meier method (log-rank test). All hypothetical tests were two-sided, and a p value < 0.05 indicated significance.

## Results

### Outline of cell types in CRC of MSI and MSS

**Figure 2:**
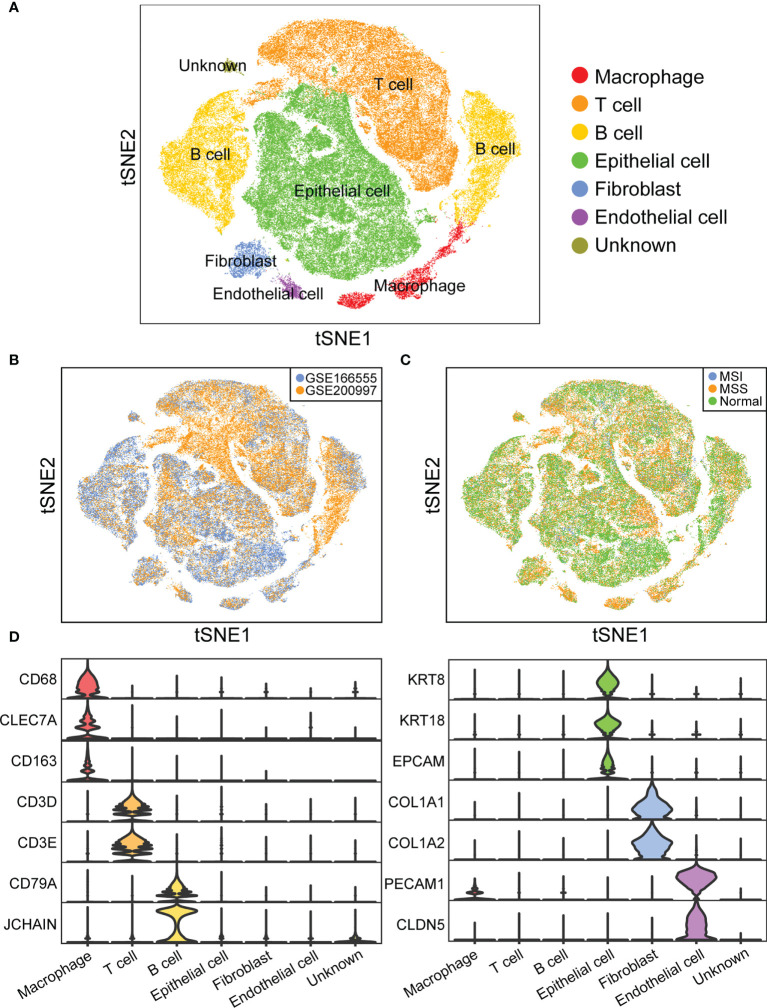
Overview of infiltrating cell types in CRC of MSI and MSS. **(A)** t-SNE plot of 111,748 cells from 46 CRC samples. **(B)** t-SNE plot showing cell distribution of GSE166555 and GSE200997. **(C)** t-SNE plot showing cell distribution of normal, cancer with MSS, and cancer with MSI samples. **(D)** Violin plot showing the expression of marker genes.

To identify the infiltrating cell types in MSI and MSS CRC, we first characterized the single-cell transcriptome atlas of tumor samples from 46 CRC samples in the GSE166555 and GSE200997 datasets. The microsatellite statuses of all the patients in the two datasets are shown in **Supplementary Table 1**. Among all 46 samples, the microsatellite status of 3 samples was MSI. The proportion of samples with MSI (6.5%) was consistent with data from many colorectal cancer cohorts (4.5%/8.7%) (39, 40). After quality control, 44,848 cells from normal samples, 57,780 cells from MSS samples, 9,120 cells from MSI samples, for a total of 111,748 cells were selected for subsequent downstream analysis. Six major cell clusters were identified and visualized using t-SNE plots (**Figure 2A**). We then evaluated the batch effect of the integrated data in the two datasets (**Figure 2B**) and the overall condition of cell clusters in the normal, cancer with MSS, and cancer with MSI samples (**Figure 2C**). Each cluster of cells was manually annotated using currently known cell markers (**Figure 2D**).

### IDO1 macrophages are more abundant in colorectal cancer with MSI

**Figure 3:**
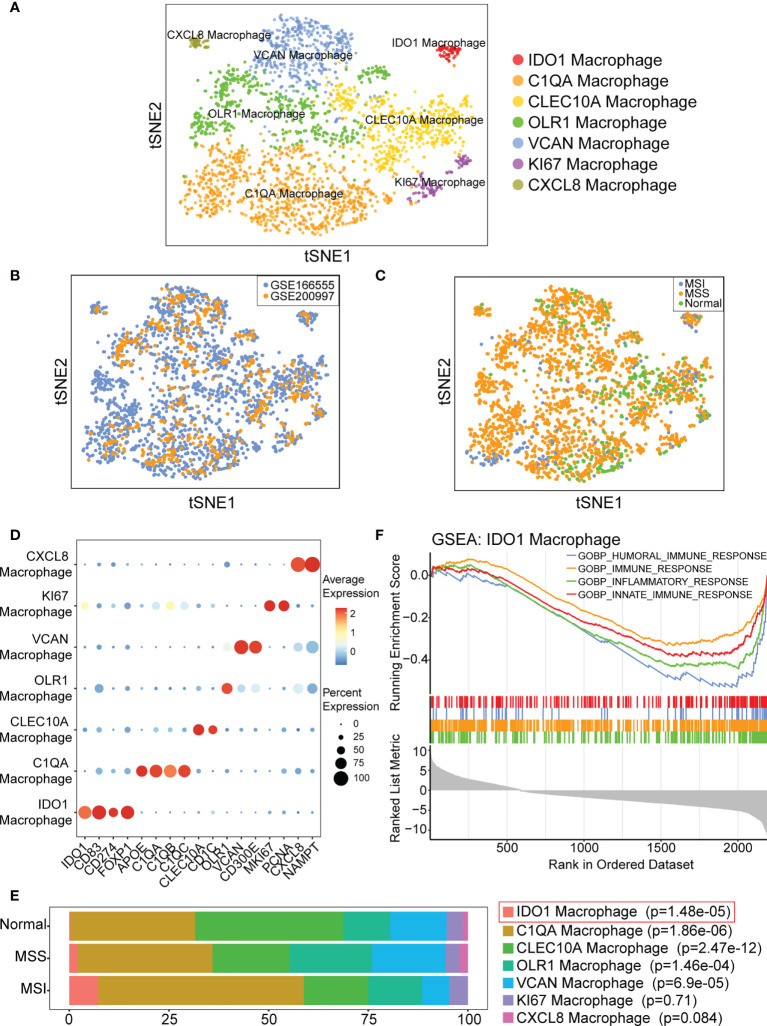
IDO1 macrophage is identified and may be associated with immunity. **(A)** t-SNE plot showing classification of macrophage subpopulations. **(B)** t-SNE plot showing distribution of macrophages from GSE166555 and GSE200997. **(C)** t-SNE plot showing distribution of macrophages in normal, cancer with MSS and cancer with MSI samples. **(D)** Bubble diagram shows the expression of subgroup marker genes. **(E)** IDO1 macrophage is predominant in CRC with MSI. p-values are calculated by chi-square test, and Fisher’s exact test is used when required. **(F)** GSEA indicates IDO1 macrophage is negatively correlated with GO terms of immune.

Macrophages clustered into seven subgroups (**Figure 3A**). We evaluated the batch effect of the integrated data in two datasets and the overall condition of macrophage clusters in the normal, cancer with MSS, and cancer with MSI samples (**Figures 3B, C**). We annotated each subgroup of macrophages according to the identified cell markers (**Figure 3D**) and identified seven specific macrophage subgroups (IDO1, C1QA, CLEC10A, OLR1, VCAN, KI67, and CXCL8 macrophages). We then compared the proportions of macrophage subgroups across the normal, MSI, and MSS samples, and the results of statistical analysis were shown in **Supplementary Table 2**. We found that the proportion of IDO1 macrophages was significantly higher (p=1.48 e-05) in CRC with MSI than in CRC with MSS or normal colorectal tissues (**Figure 3E**). Moreover, IDO1 macrophage subset was different from other macrophage subsets, which was the only macrophage subset presented almost exclusively in colorectal cancer tissue. The results indicated that IDO1 macrophages were closely related to colorectal cancer. To better understand the function of IDO1 macrophages, enrichment analysis was performed. The results of GSEA indicated that IDO1 macrophages were negatively correlated with gene ontology (GO) terms of immunity, including humoral, immune, inflammatory, and innate immune responses (**Figure 3F**).

### Cell-cell interaction analysis of IDO1 macrophages

**Figure 4:**
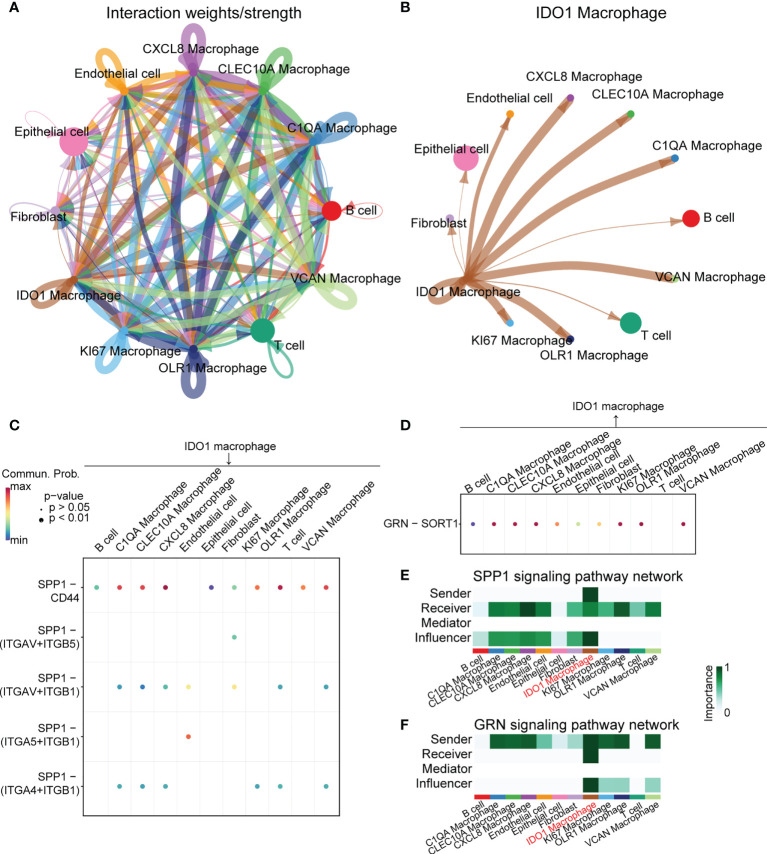
Cell-cell interaction analysis of IDO1 macrophage. **(A)** Overall communication condition of all cell clusters. Circle sizes are proportional to the number of cells in each cell group and edge width represents the communication probability. **(B)** Communication condition between IDO1 macrophage and other cell clusters. Circle sizes are proportional to the number of cells in each cell group and edge width represents the communication probability. **(C, D)** Signaling pathways that mainly contain IDO1 macrophages are identified. **(E)** IDO1 macrophage is the unique sender in the SPP1 signaling pathway network. **(F)** IDO1 macrophage is the unique receiver in the GRN signaling pathway network.

Cell communication analysis was performed using CellChat, and signal networks related to IDO1 macrophages were identified. **Figure 4A** shows the overall communication conditions for all cell clusters. IDO1 macrophages may interact with many cells, including T cells and B cells (**Figure 4B**). We showed the outgoing and incoming signals based on the IDO1 macrophages (**Supplementary Figure 1**). Signaling pathways that mainly involved IDO1 macrophages were identified (**Figures 4C, D**). In signaling pathways, the term “sender” refers to a signaling source, “receiver” refers to a signaling target, “mediator” refers to a gatekeeper of cell-cell communication, and “influencer” refers to a component that has the ability to influence information flow within a signaling network. IDO1 macrophages were unique senders in the SPP1 signaling network and unique receivers in the granulin (GRN) signaling network (**Figures 4E, F**).

### Identification of genes related to IDO1 macrophages in MSI-H state and construction of GSVA score

**Figure 5:**
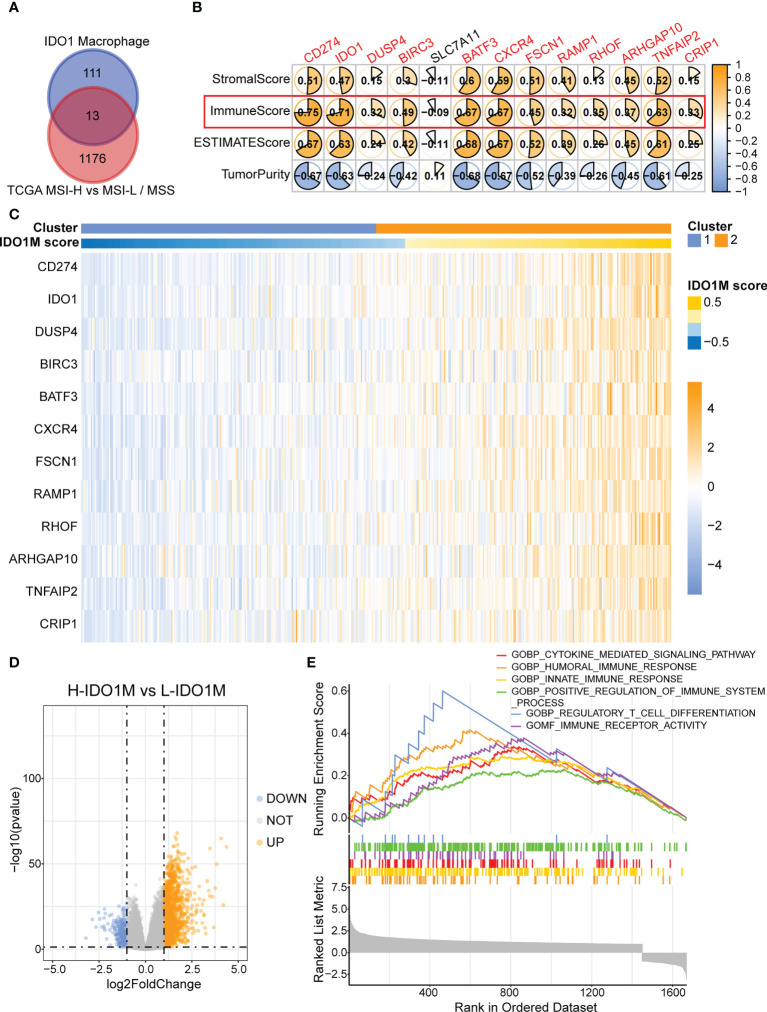
Construction of IDO1M score and clustering of IDO1M. **(A)** Venn diagram shows the intersection of MSI-H and IDO1 macrophages marker genes. **(B)** Correlation between 13 genes and immune parameters mainly including immune score. **(C)** Heatmap shows the clustering of patients based on IDO1M score. **(D)** Volcano plot of differential analysis between H-IDO1M and L-IDO1M. **(E)** GSEA shows many immunological terms are significantly enriched in H-IDO1M.

To accurately identify genes associated with IDO1 macrophages, we combined the scRNA-seq and bulk-seq data. We performed a differential analysis of TCGA-COAD patients between the MSI-H (n = 78) and MSS (n = 269)/MSI-L (n = 79) groups. We identified 1,189 upregulated genes in patients with MSI-H. We then intersected these genes with those that were highly expressed in IDO1 macrophages and identified 13 such genes (**Figure 5A**). We then performed Spearman’s correlation analysis between gene expression and various immune parameters—primarily the immune score (**Figure 5B**). We set a threshold immunity score of > 0.3 and retained 12 eligible genes. We then utilized the R package GSVA to calculate IDO1M scores for each sample in TCGA-COAD, regardless of microsatellite status, and divided the samples into L-IDO1M and H-IDO1M clusters based on the median scores (**Figure 5C**).

### Comparison of functional and clinical characteristics

To better understand the differences between the two clusters, differential analysis was performed, which revealed 1,454 upregulated and 225 downregulated genes in H-IDO1M (**Figure 5D**). GSEA was used to compare the functional differences between the two clusters (**Figure 5E**). Many immunological terms were significantly enriched in H-IDO1M, including the cytokine-mediated signaling pathway, humoral immune response, innate immune response, positive regulation of the immune system, regulatory T cell differentiation, and immune receptor activity. Subsequently, the clinical characteristics of these patients were compared. A chi-squared test showed that H-IDO1M had a lower pathological stage and a larger proportion of female patients than L-IDO1M (**Table 1**). There was no difference in prognosis between the two clusters (**Supplementary Figure 2**). Therefore, infiltration of IDO1 macrophages may influence the immune system and inhibit tumor metastasis.

**Table 1:** Comparison of clinical characteristics between L-IDO1M cluster and H-IDO1M cluster.

	L-IDO1M	H-IDO1M	P value
Number	185	184	
Age [median (IQR)]	68.00 [58.00, 75.00]	69.00 [59.75, 78.00]	0.139
Gender (%)			0.014*
female	75 (40.5)	99 (53.8)	
male	110 (59.5)	85 (46.2)	
T stage (%)			0.59
T1/T2	36 (19.5)	41 (22.3)	
T3/T4	149 (80.5)	143 (77.7)	
N stage (%)			0.153
N0	99 (53.5)	113 (61.4)	
N1/N2	86 (46.5)	71 (38.6)	
M stage (%)			0.059
M0	131 (79.4)	149 (87.6)	
M1	34 (20.6)	21 (12.4)	
Pathological stage (%)			0.04*
Stage I/II	93 (50.3)	113 (61.4)	
Stage III-IV	92 (49.7)	71 (38.6)	

(*: P < 0.05).

### Comparison of immune infiltration between two clusters

**Figure 6:**
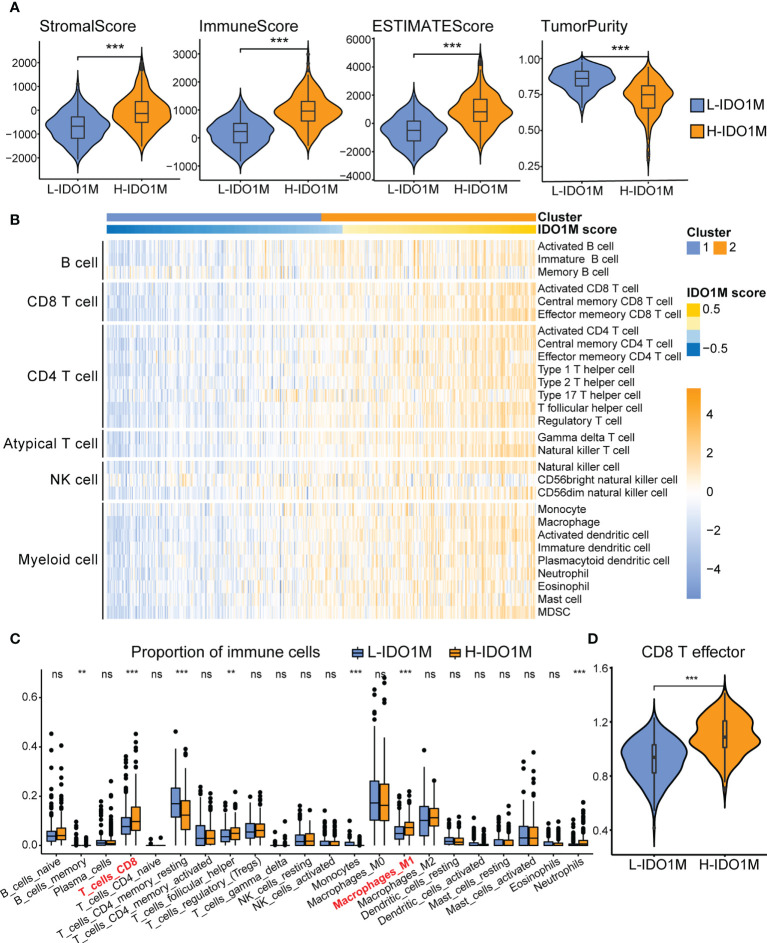
Comparison of immune infiltration between H-IDO1M and L-IDO1M in TCGA-COAD. **(A)** The ESTIMATE analysis shows that H-IDO1M has higher stromal, immune, and ESTIMATE scores but lower tumor purity than L-IDO1M. **(B)** ssGSEA shows H-IDO1M has a higher level of immune cell infiltration than L-IDO1M. **(C)** CIBERSORT analysis demonstrates that H-IDO1M has a higher proportion of CD8 T cells and M1 macrophages. **(D)** ssGSEA shows H-IDO1M has a higher level of CD8 T effector than L-IDO1M. (ns, no significance, **P < 0.01, ***: P < 0.001).

ESTIMATE, ssGSEA, and CIBERSORT were used to explore differences in immunological function. H-IDO1M had higher stromal, immune, and ESTIMATE scores and lower tumor purity than L-IDO1M in the ESTIMATE analysis (**Figure 6A**). Furthermore, ssGSEA showed that the level of immune cell infiltration in H-IDO1M was higher than that in L-IDO1M (**Figure 6B**). Next, we determined the proportion of immune cells in these two clusters. CIBERSORT analysis demonstrated that H-IDO1M cells had a higher proportion of CD8+ T cells and M1 macrophages (**Figure 6C**). In addition, ssGSEA showed that the level of CD8 T effector in H-IDO1M was higher than that in L-IDO1M (**Figure 6D**). These results indicate that H-IDO1M exhibit stronger immune infiltration than L-IDO1M, especially CD8+ T cells and M1 macrophages.

### Predictive assessment of response to immunotherapy

**Figure 7:**
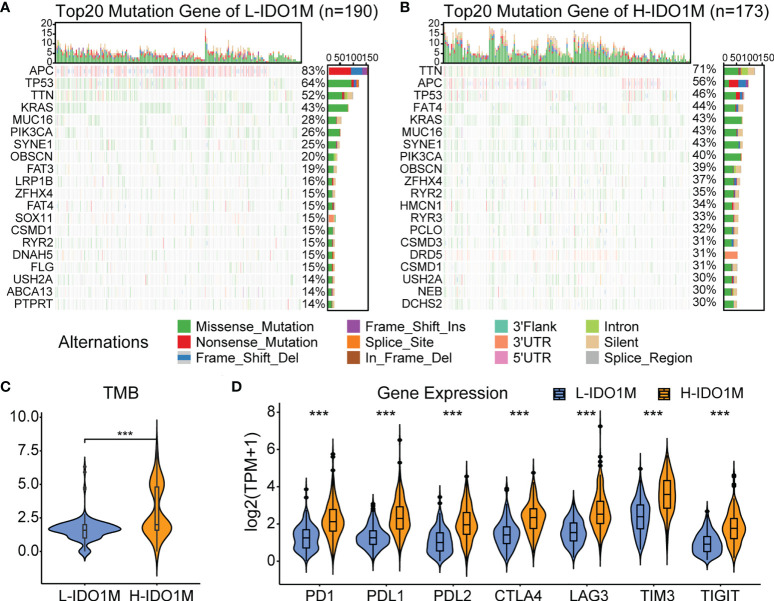
Evaluation of response to immunotherapy in TCGA-COAD. **(A)** Landscapes of mutation profiles of L-IDO1M cluster. **(B)** Landscapes of mutation profiles of H-IDO1M cluster. **(C)** Comparison of TMB levels in two clusters. **(D)** Comparison of the expression of immune checkpoints in two clusters. (***: P < 0.001).

**Figure 8:**
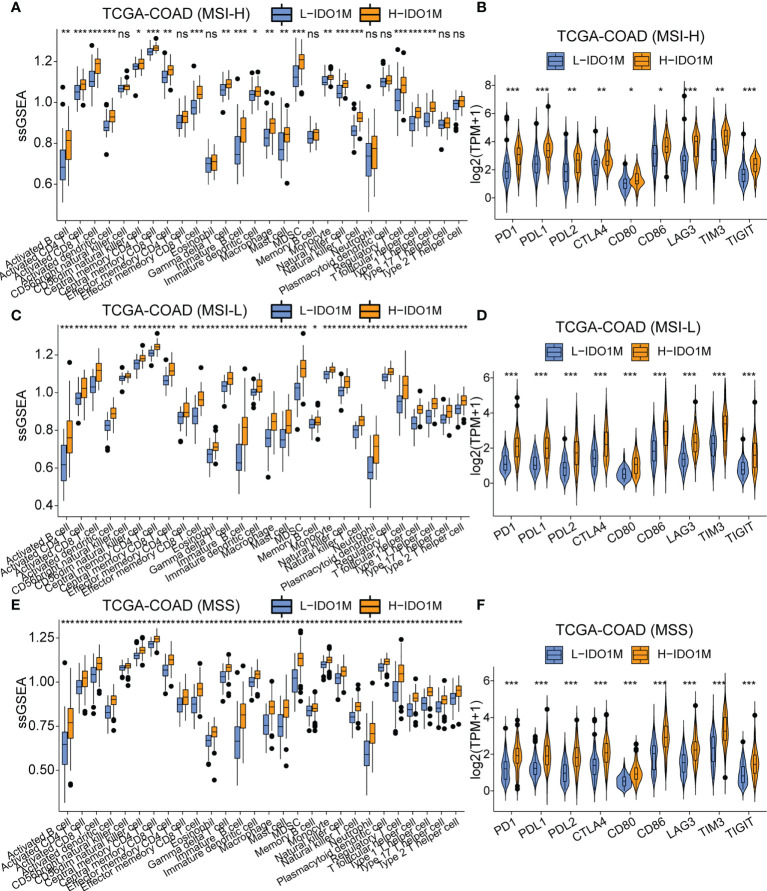
Comparison of immune infiltration and the expression of immune checkpoints between H-IDO1M and L-IDO1M in patients with MSI-H, MSI-L, and MSS. **(A, C, E)** ssGSEA shows H-IDO1M has a higher level of immune cell infiltration than L-IDO1M. **(B, D, F)** Comparison of the expression of immune checkpoints in two clusters. (ns, no significance, *: P < 0.05, **: P < 0.01, ***: P < 0.001).

**Figure 9:**
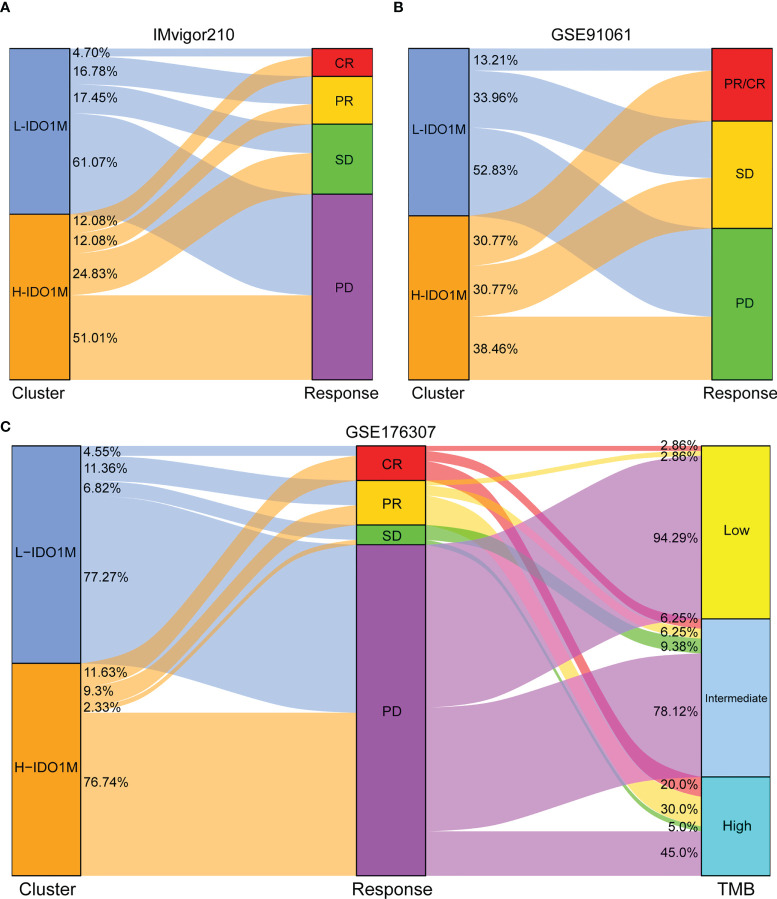
Evaluation of response to immunotherapy in two immunotherapy cohorts. **(A, B)** Sankey diagram shows the relationship between IDO1M scores and response to immunotherapy in patients from IMvigor210 and GSE91061. **(C)** Sankey diagram shows the relationship between IDO1M scores/TMB and response to immunotherapy in patients from GSE176307.

To evaluate the response to immunotherapy, we first compared the landscape of the mutation profiles of the two clusters (**Figures 7A, B**). We then calculated the TMB of each sample and found that H-IDO1M had a higher TMB than L-IDO1M (**Figure 7C**), which may have led to the production of more neoantigens to stimulate an immune response. We compared the expression of immune checkpoints between the two clusters and found that proteins functioning at important immune checkpoints (PD1, PDL1, PDL2, CTLA4, LAG3, TIM3, and TIGHT) were significantly upregulated in H- IDO1M (**Figure 7D**). To assess the effect of IDO1M scores in predicting the response to immunotherapy in patients with different microsatellite statuses (MSI-H, MSI-L, and MSS), we calculated the levels of immune cell infiltration and immune checkpoint expression in each sample of these three groups separately. The results showed that patients in each group with high IDO1M scores had higher immune infiltration and immune checkpoint expression (**Figure 8**). We then looked for any correlation between IDO1M scores and immunotherapeutic response (IMvigor210 and GSE91061). IMvigor210 is a bladder cancer cohort treated with PDL1. GSE91061 is a melanoma cohort treated with CTLA4 and PD1. Immunotherapeutic responses were divided into four categories: complete response (CR), partial response (PR), stable disease (SD), and progressive disease (PD). In IMvigor210, H-IDO1M had a higher CR and lower PD than L-IDO1M (**Figure 9A**). In GSE91061, H-IDO1M had a higher CR and PR and lower PD than L-IDO1M (**Figure 9B**). Moreover, we also utilized TMB as a predictor of response to immunotherapy (GSE176307). GSE176307 is a urothelial cancer cohort treated with PD1/PDL1. In GSE176307, H-IDO1M had a higher CR than L-IDO1M, and high-TMB had a higher CR and PR and lower PD than intermediate-TMB as well as low-TMB (**Figure 9C**). The results indicate that H-IDO1M respond better to immunotherapy than L-IDO1M, and IDO1M scores and TMB have similar trends in predicting immunotherapy response.

### GEO verification between two clusters

**Figure 10:**
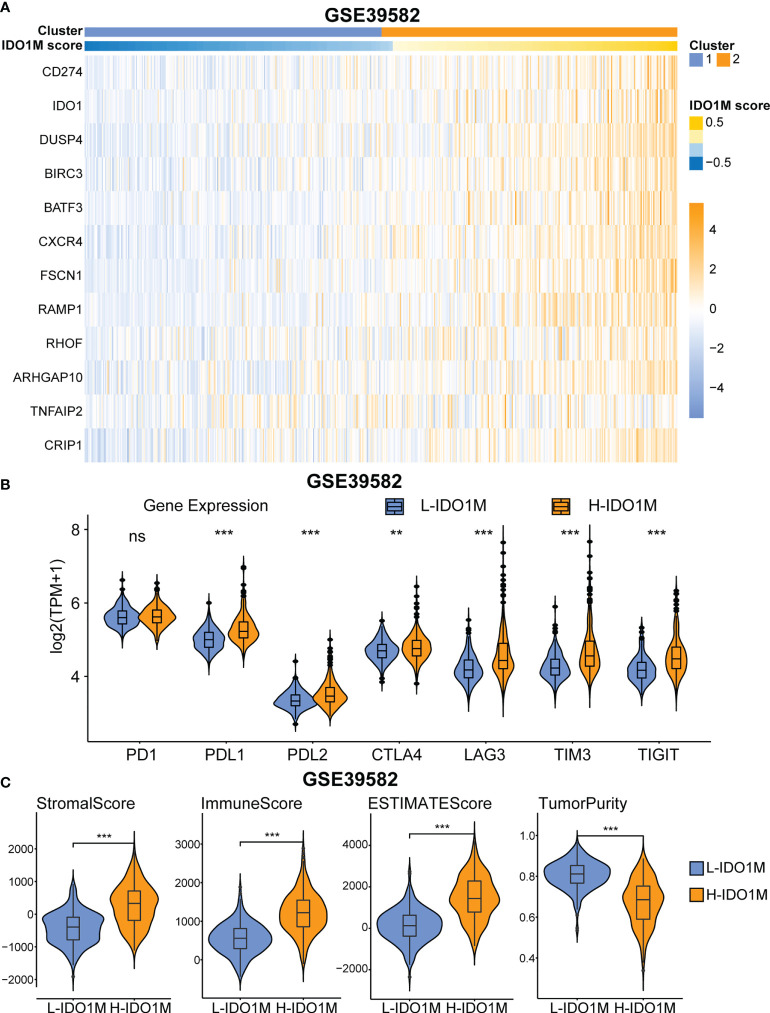
GEO validation of immune characteristics between two clusters. **(A)** Heatmap shows the expression pattern of IDO1M-related genes in two clusters. **(B)** The expression of all immune checkpoints except PD1 is high in H-IDO1M. **(C)** The ESTIMATE analysis shows that H-IDO1M has higher stromal, immune, and ESTIMATE scores but lower tumor purity than L-IDO1M. (ns, no significance, **: P < 0.01, ***: P < 0.001).

**Figure 11:**
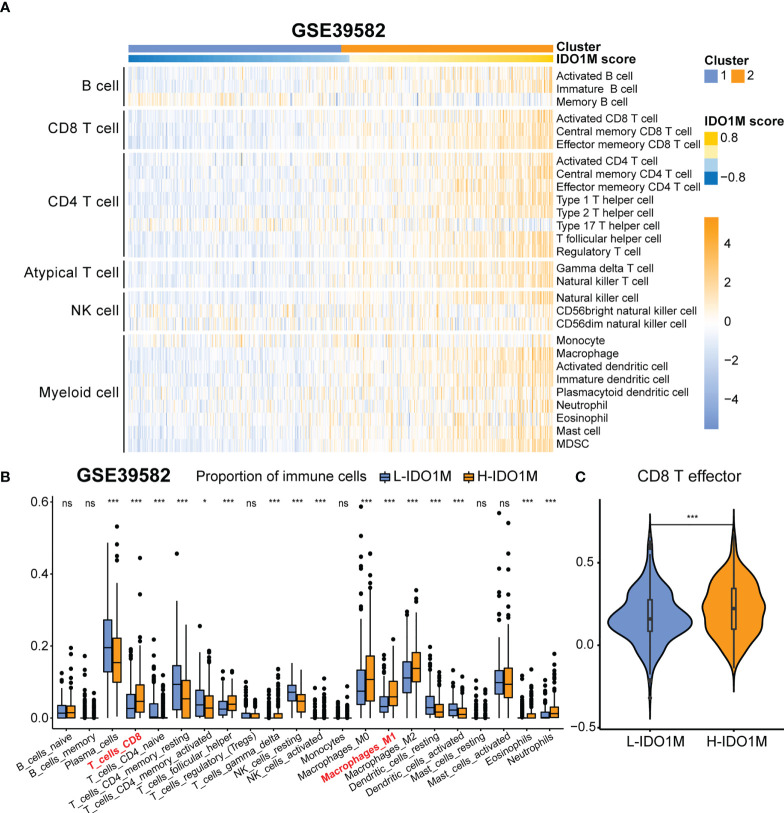
Comparison of immune infiltration between H-IDO1M and L-IDO1M in GSE39582. **(A)** ssGSEA shows H-IDO1M has a higher level of immune cell infiltration than L-IDO1M. **(B)** CIBERSORT analysis demonstrates that H-IDO1M has a higher proportion of CD8 T cells and M1 macrophages. **(C)** ssGSEA shows H-IDO1M has a higher level of CD8 T effector than L-IDO1M. (ns, no significance, *: P < 0.05, ***: P < 0.001).

To further verify the correlation between IDO1M scores and immunotherapeutic response, we calculated the IDO1M scores of each sample and divided the 519 colon cancer samples (dMMR=75, pMMR=444) from GSE39582 into two clusters, as performed in TCGA (**Figure 10A**). The expression of immune checkpoints and extent of immune infiltration (ESTIMATE, ssGSEA, and CIBERSORT) were evaluated in the same manner as TCGA-COAD. The expression of all immune checkpoints except PD1 was high in the H-IDO1M group (**Figure 10B**). The results of immune cell infiltration were consistent with those of TCGA-COAD (**Figures 10C** and **Figures 11A–C**). Therefore, using GSE39582, we verified that H-IDO1M may contribute to a more active immune system and thus a better response to immunotherapy than L-IDO1M. Then, we calculated the levels of immune cell infiltration and immune checkpoint expression in each sample of these three groups separately. The results showed that patients in each group with high IDO1M scores had higher immune infiltration and immune checkpoint expression (**Supplementary Figure 3**).

## Discussion

Macrophages have been shown to play an essential role in influencing the development and metastasis of CRC, but their specific role remains controversial due to their complex TME (41–44). scRNA-seq helps us identify distinct macrophage subsets in this complex TME, providing new insights for immunotherapy. Using scRNA-seq, we identified a subset of macrophages with high IDO1 expression. IDO1, which is induced by interferon-γ, is highly expressed in CRC and is associated with the promotion of CRC cell proliferation and inhibition of apoptosis (45, 46). IDO1 has been implicated in mediating immunosuppression in cancer, and high expression of IDO1 in CRC cells can counteract T cell invasion through tryptophan depletion and production of proapoptotic tryptophan catabolites (47, 48). However, whether macrophages with high IDO1 expression affect tumor immunity remains unclear. In this study, we integrated scRNA-seq and bulk-seq to analyze the role of IDO1 macrophages in patients with CRC. Our results demonstrate that the proportion of IDO1 macrophages in patients with MSI was higher than that in patients with MSS. Unlike other macrophage subsets, IDO1 macrophages were almost absent in normal colorectal tissue, suggesting that IDO1 macrophages are closely associated with CRC. Moreover, GSEA revealed that IDO1 macrophages were associated with immune suppression. We explored the cell communication and signaling pathways associated with IDO1 macrophages using CellChat. Our results indicated that IDO1 macrophages are unique senders in the SPP1 signaling network and unique receivers in the GRN signaling network. These results imply that IDO1 macrophages might act as a signaling source in the SPP1 signaling pathway, transmitting the signal to cells where the receptor is located, and that SPP1 receptors such as CD44 can receive the signal and produce a specific cellular response. These results also implied that IDO1 macrophages might serve as a signaling target of the GRN signaling pathway, and receptors of GRN, such as SORT1, might be localized on IDO1 macrophages to receive upstream signal transmission, thereby producing a specific cellular response. SPP1 is associated with TAM and can trigger the polarization of macrophages to M2-phenotype TAMs by upregulating the expression of PD-L1 (49, 50). Granulin (GRN) is highly expressed in multiple tumors and can restore the infiltration of CD8+ T cells in pancreatic ductal adenocarcinoma, indicating that immunotherapy targeting macrophage-derived GRN is promising (51).

We identified markers of IDO1 macrophages using scRNA-seq and highly expressed genes in COAD patients with MSI using bulk-seq. We then considered the intersection of the results obtained by these two analytical methods and performed a correlation analysis using a series of immunological parameters for duplicate genes. Ultimately, we identified 12 genes associated with IDO1 macrophages that were related to both MSI and immunity (CD274, IDO1, DUSP4, BIRC3, BATF3, CXCR4, FSCN1, RAMP1, RHOF, ARHGAP10, TNFAIP2, and CRIP1). CD274, also known as programmed cell death ligand 1 (PD-L1), is an immune checkpoint molecule expressed in tumor cells that binds to programmed death 1 (PD1) in T cells (52). PD-L1 expression in tumor cells enables immune evasion by inhibiting CD8+ T cell cytotoxicity (53). DUSP4 (MKP-2) is a member of the mitogen-activated protein kinase phosphatase (MKP) family that inhibits the proliferation of CD4+ T cells by regulating the phosphorylation of STAT5 (54). BIRC3 is an inhibitor of apoptosis protein (IAP), and its expression is regulated by tumor necrosis factor alpha (TNF alpha). Its upregulation in tumor cells inhibits the activity of natural killer (NK) cells to kill tumors (55, 56). BATF3 is a transcription factor that promotes proliferation, invasion, and metastasis of CRC cells (57). Moreover, BATF3 can influence the apoptosis and longevity of T cells *via* the proapoptotic factor BIM, indicating that BATF3 has the potential to optimize adoptive T-cell therapy (ACT) in cancer patients (58). CXCR4 is a G-protein-coupled receptor that binds its ligand, CXCL12. This complex can activate multiple signaling pathways and regulate cancer stem cells; therefore, effective tumor immunotherapy targeting CXCR4-CXCL12 is crucial (59, 60). Fascin actin-bundling protein 1 (FSCN1) is highly expressed in multiple tumors and is associated with the progression of CRC and lung adenocarcinoma, and poor prognosis in adrenocortical carcinoma (61–63). RHOF is a member of the Rho GTPase family and promotes marginal zone (MZ) B cell development in the spleen (64).

We constructed IDO1M scores using GSVA and divided the samples into L-IDO1M and H-IDO1M clusters based on the median of the IDO1M scores. We then evaluated their clinical characteristics and found that H-IDO1M had a lower pathological stage than L-IDO1M, suggesting that the IDO1M scores may be related to CRC progression. We then performed differential analysis and applied GSEA to identify functional differences between the two clusters. GSEA indicated that immune-related pathways were enriched in H-IDO1M, including cytokine-mediated signaling, humoral immune response, innate immune response, positive regulation of the immune system, regulatory T cell differentiation, and immune receptor activity.

To better understand the differences in immunological function between the H-IDO1M and L-IDO1M clusters, we utilized ESTIMATE, ssGSEA, and CIBERSORT. The ESTIMATE results indicated that the H-IDO1M cluster had higher stromal, immune, and ESTIMATE scores than the L-IDO1M cluster. ssGSEA results showed that the level of immune cell infiltration in the H-IDO1M cluster was higher than that in the L-IDO1M cluster. Furthermore, CIBERSORT analysis demonstrated that the H-IDO1M cluster had a higher proportion of CD8+ T cells and M1 macrophages than the L-IDO1M cluster. CD8+ tumor-infiltrating lymphocytes (TILs) mediate tumor rejection by recognizing tumor antigens (65). Moreover, M1 macrophages exert antitumor functions, including direct mediation of cytotoxicity and antibody-dependent cell-mediated cytotoxicity (ADCC) to kill tumor cells (66). These analyses suggest that the H-IDO1M cluster exhibits higher immune infiltration than the L-IDO1M cluster, especially CD8+ T cells and M1 macrophages, which might be a favorable condition for immunotherapy in patients with CRC. The underlying reason may be the heterogeneity of tumor immunophenotyping. Human tumors can be categorized as having an inflamed, immune desert, or immune-excluded phenotypes based on the complexities of immune infiltration (67). The degree of immune cell infiltration is generally higher in inflamed tumors and lower in immune desert tumors and there is a positive correlation between the infiltration of immune cells in bulk-seq data. Therefore, in tumor tissue performed bulk-seq, the infiltration of IDO1 macrophages may be accompanied by the infiltration of other immune cells, such as CD8+ T cells and M1 macrophages, resulting in H-IDO1M scores associated with immune infiltration and immunotherapy response.

To evaluate the impact of IDO1M scores on immunotherapy, we compared the mutation profiles of the two clusters. The results indicated that the H-IDO1M cluster had a significantly higher overall mutation rate than that of the L-IDO1M cluster. We then calculated the TMB of each sample and found that the H-IDO1M cluster had higher TMB than the L-IDO1M cluster. Neoantigens are produced as a result of mutations. Thus, a higher TMB results in more neoantigens, increasing chances for T cell recognition and correlating clinically with better outcomes of immunotherapy (68). In addition, we assessed the expression of classical immune checkpoints (PD1, PDL1, PDL2, CTLA4, LAG3, TIM3, and TIGHT) between the two clusters (69). The high expression of immune checkpoints is a favorable condition for the application of immune checkpoint inhibitors (ICIs), which is beneficial for immunotherapy (70, 71). The H-IDO1M cluster in each microsatellite status (MSI-H, MSI-L, and MSS) had a higher level of immune cell infiltration and immune checkpoint expression, indicating that H-IDO1M scores can predict responses to immunotherapy independent of microsatellite status. We then evaluated the effect of the IDO1M scores in the two immunotherapeutic cohorts. We found that in IMvigor210, H-IDO1M had a higher CR and lower PD than L-IDO1M, and in GSE91061, H-IDO1M had a higher CR and PR and lower PD than L-IDO1M. These results indicate that H-IDO1M have a better response to immunotherapy than L-IDO1M.

To further validate the impact of the IDO1M scores on immunotherapy, we divided the samples from GSE39582 into two clusters based on the IDO1M scores. As in TCGA, we explored the expression of immune checkpoints and degree of immune infiltration. The results showed that the expression levels of most immune checkpoints in the H-IDO1M cluster were high. In addition, the immune infiltration results were consistent with those of TCGA-COAD. Therefore, GSE39582 further validated that H-IDO1M may have a better immunotherapy response than L-IDO1M.

Although the combined analysis of scRNA-seq and bulk-seq has helped us understand that IDO1 macrophages may be associated with immunotherapy in CRC, further mechanistic studies are required. This study had some limitations. First, this was a retrospective study and prospective studies should be considered to avoid analytical bias. Second, this study is based on transcriptomics; however, both proteomic and spatial transcriptomic data are worth exploring. Third, because of the lack of information on the microsatellite/MMR status of patients in immunotherapy cohorts, it is hard to compare the predictive ability of MSI-H and IDO1M scores for tumor immunotherapy response.

In conclusion, our study identified IDO1 macrophages, which were abundant in patients with CRC who exhibit MSI. We then identified 12 genes related to IDO1 macrophages and determined IDO1M scores for each sample to predict the response to immunotherapy in COAD patients. H-IDO1M exhibited a higher extent of immune infiltration, TMB, expression of immune checkpoints, and a better response to immunotherapy than L-IDO1M. These findings provide new insights into tumor immunity and may be useful clinically when designing appropriate immunotherapy for patients with COAD.

## Data availability statement

The datasets presented in this study can be found in online repositories. The names of the repository/repositories and accession number(s) can be found in the article/**Supplementary Material**.

## Author contributions

XL designed the study and wrote the manuscript. GY performed the literature search and collected data for the manuscript. BX analyzed the data. HY edited the figures and tables. MS and YA revised the manuscript. All authors contributed to the article and approved the submitted version.

## Acknowledgments

All authors would like to thank the sample donors and research teams for the TCGA, GSE166555, GSE200997, GSE39582, GSE91061 and IMvigor210 cohort which provided data for this article.

## Conflict of interest

The authors declare that the research was conducted in the absence of any commercial or financial relationships that could be construed as a potential conflict of interest.

## Publisher’s note

All claims expressed in this article are solely those of the authors and do not necessarily represent those of their affiliated organizations, or those of the publisher, the editors and the reviewers. Any product that may be evaluated in this article, or claim that may be made by its manufacturer, is not guaranteed or endorsed by the publisher.
