# Supplementary material for: Evaluating the role of IDO1 macrophages in immunotherapy using scRNA-seq and bulk-seq in colorectal cancer
Source: Front Immunol. 2022 Sep 29;13:1006501. doi: 10.3389/fimmu.2022.1006501 (PMC9556727; doi:10.3389/fimmu.2022.1006501)
Supplement: Supplementary file 1 [file DataSheet_1.docx]

Supplementary Material

**Supplementary Figure 1.** Outgoing and incoming signals based on IDO1 macrophages. (A) Outgoing signals from IDO1 macrophages. (B) Incoming signals to IDO1 macrophages. Dot color reflects communication probabilities and dot size represents computed p-values. Empty space means the communication probability is zero.

**Supplementary Figure 2.** Kaplan-Meier curve for L-IDO1M cluster and H-IDO1M cluster. There is no difference in the prognosis between L-IDO1M and H-IDO1M.

**Supplementary Figure 3.** Comparison of immune infiltration and the expression of immune checkpoints between H-IDO1M and L-IDO1M in patients with dMMR and pMMR. (A,C) ssGSEA shows H-IDO1M has a higher level of immune cell infiltration than L-IDO1M. (B,D) Comparison of the expression of immune checkpoints in two clusters.

**Supplementary Table 1.** The microsatellite status of patients in GSE166555 and GSE200997.

**Supplementary Table 2.** Comparison of the proportion of macrophage subsets among normal, MSS, and MSI samples.
