# Supplementary figures and images for: Evaluating the role of IDO1 macrophages in immunotherapy using scRNA-seq and bulk-seq in colorectal cancer
Source: Front Immunol. 2022 Sep 29;13:1006501. doi: 10.3389/fimmu.2022.1006501 (PMC9556727; doi:10.3389/fimmu.2022.1006501)

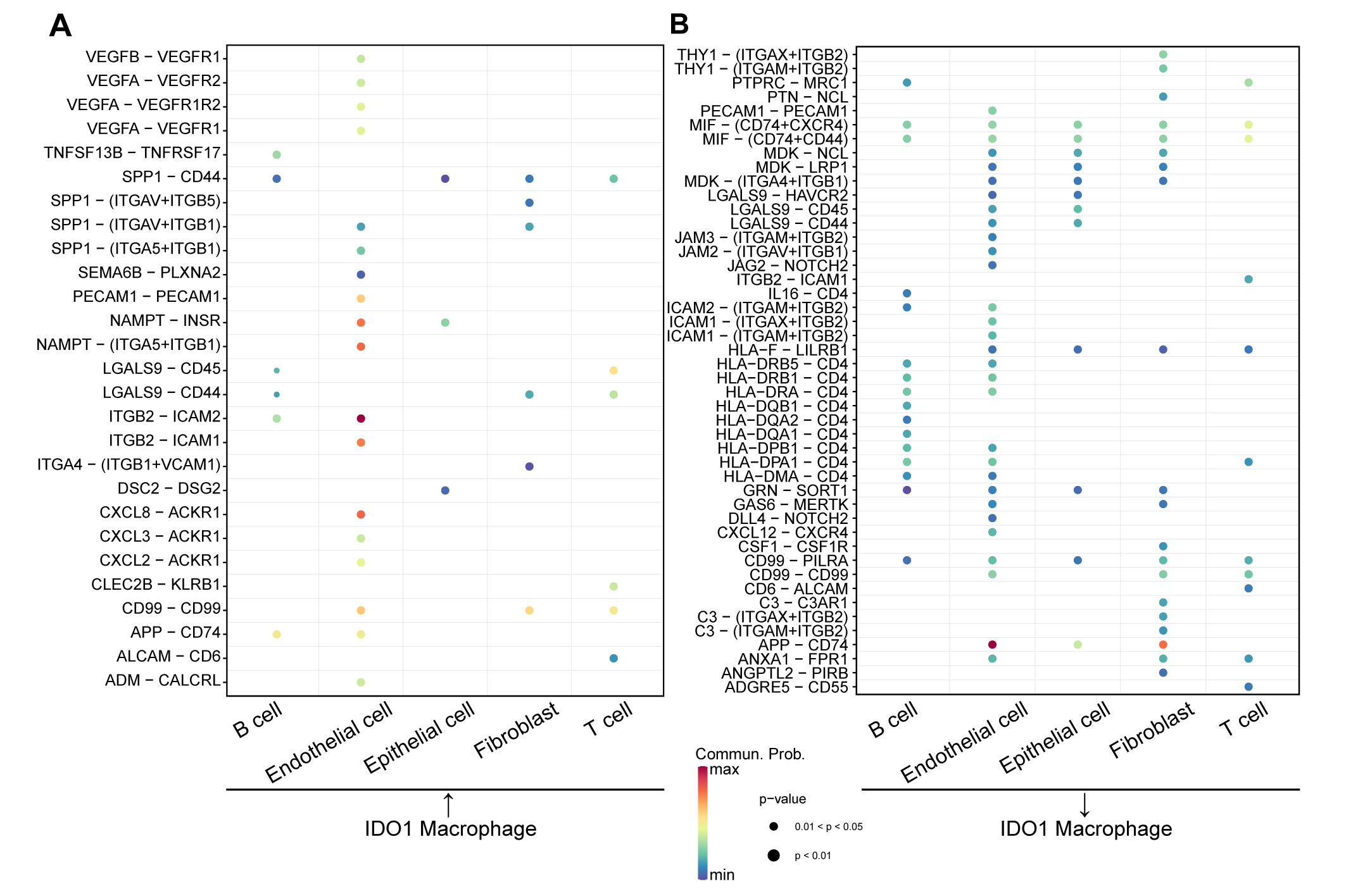

Supplement: Supplementary file 4 [file Image_1.tif]

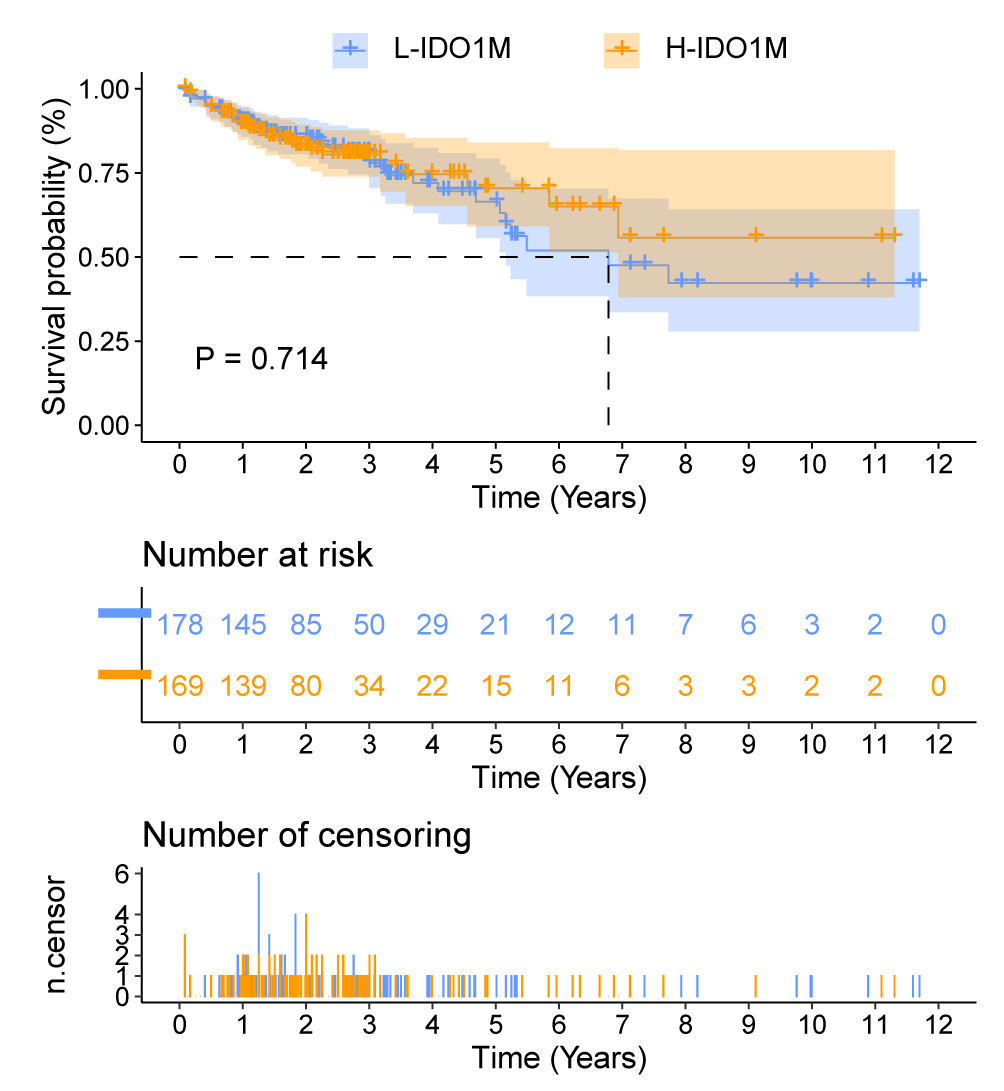

Supplement: Supplementary file 5 [file Image_2.tif]

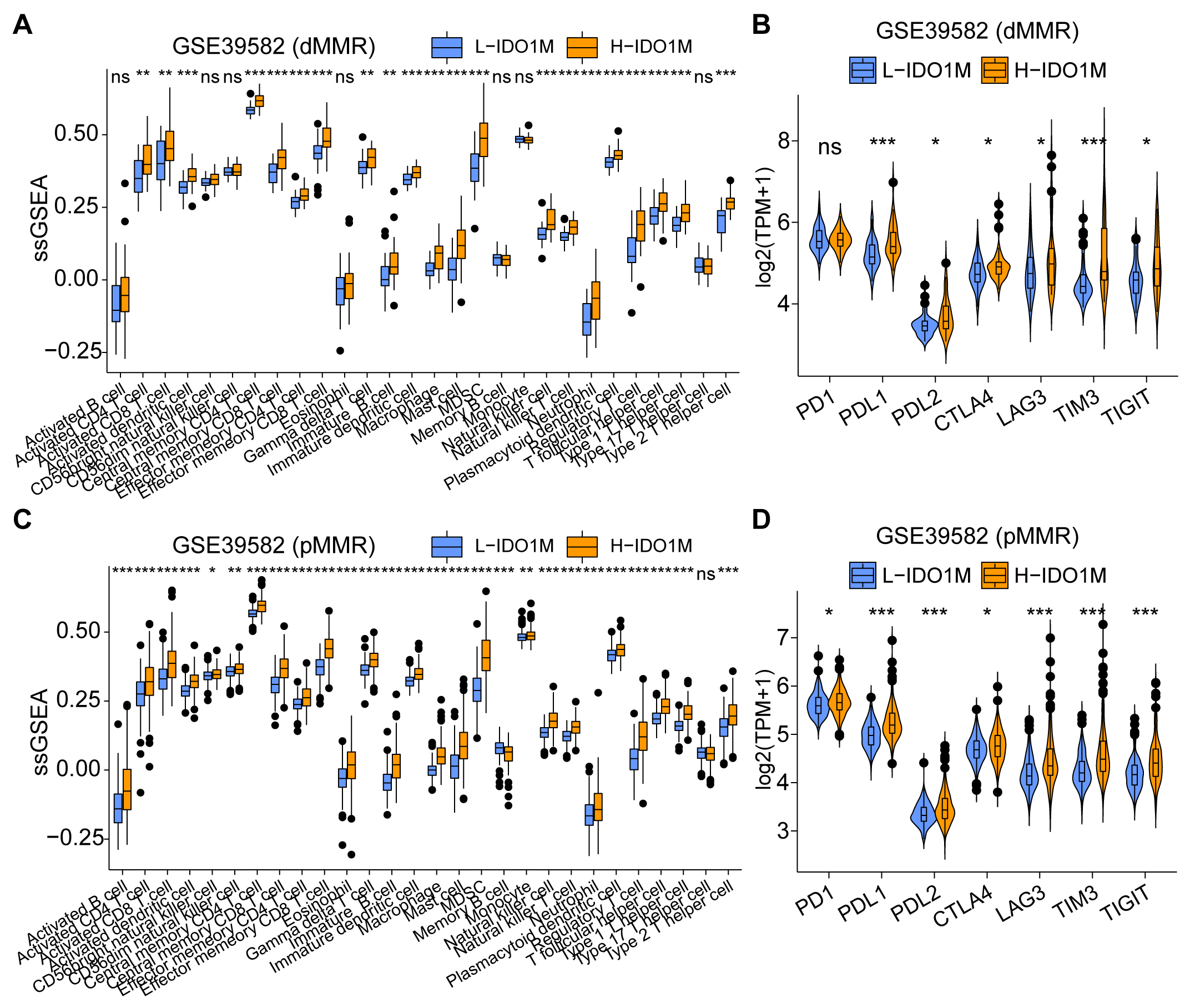

Supplement: Supplementary file 6 [file Image_3.tif]
